# Supporting children to adhere to anti-retroviral therapy in urban Malawi: multi method insights

**DOI:** 10.1186/1471-2431-9-45

**Published:** 2009-07-14

**Authors:** Ralf Weigel, Ireen Makwiza, Jean Nyirenda, Darles Chiunguzeni, Sam Phiri, Sally Theobald

**Affiliations:** 1 The Lighthouse Trust at Kamuzu Central Hospital, PO Box 106, Lilongwe, Malawi; 2 REACH Trust (Research for Equity and Community Health), PO Box 1597, Lilongwe, Malawi; 3 UNC (University of North Carolina) Project Lilongwe, Private Bag A104, Lilongwe, Malawi; 4 International Health Research Group, Liverpool School of Tropical Medicine, Pembroke Place, Liverpool, L3 5QA, UK

## Abstract

**Background:**

Ensuring good adherence is critical to the success of anti-retroviral treatment (ART). However, in resource-poor contexts, where paediatric HIV burden is high there has been limited progress in developing or adapting tools to support adherence for HIV-infected children on ART and their caregivers. We conducted formative research to assess children's adherence and to explore the knowledge, perceptions and attitudes of caregivers towards children's treatment.

**Methods:**

All children starting ART between September 2002 and January 2004 (when ART was at cost in Malawi) were observed for at least 6 months on ART. Their adherence was assessed quantitatively by asking caregivers of children about missed ART doses during the previous 3 days at monthly visits. Attendance to clinic appointments was also monitored. In June and July 2004, four focus group discussions, each with 6 to 8 caregivers, and 5 critical incident narratives were conducted to provide complementary contextual data on caregivers' experiences on the challenges to and opportunities of paediatric ART adherence.

**Results:**

We followed prospectively 47 children who started ART between 8 months and 12 years of age over a median time on ART of 33 weeks (2–91 weeks). 72% (34/47) never missed a single dose according to caregivers' report and 82% (327/401) of clinic visits were either as scheduled, or before or within 1 week after the scheduled appointment. Caregivers were generally knowledgeable about ART and motivated to support children to adhere to treatment despite facing multiple challenges. Caregivers were particularly motivated by seeing children begin to get better; but faced challenges in meeting the costs of medicine and transport, waiting times in clinic, stock outs and remembering to support children to adhere in the face of multiple responsibilities.

**Conclusion:**

In the era of rapid scale-up of treatment for children there is need for holistic support strategies that focus on the child, the caregiver and the health worker and which are situated within the reality of fragile health systems. The findings highlight the need for cost-free and less complex paediatric ART regimes and culturally appropriate tools to support children's adherence.

## Background

The availability of anti-retroviral therapy (ART) has led to a reduction of morbidity and mortality in people living with HIV/AIDS. The push to provide ART for HIV infected children has recently gained considerable momentum in Africa, where HIV prevalence rates are high [1,2]. For example Malawi, with a population of approximately 13 million people, had an estimated 900,000 people infected with HIV in 2007, among them 90,000 children less than 15 years. Approximately 85,000 people were newly infected with HIV and 61,000 children and adults died due to AIDS [3]. Malawi has made impressive strides in scaling up access to ART. By 30^th ^September 2008, 208,440 patients (8% children) had been registered for ART in public and private clinics and 66% of those are alive and on ART [4].

In line with the equity in ART policy, ART has been provided free on a first come – first served basis since June 2004. However, using HIV prevalence rates amongst blood donors to estimate national prevalence, it was found that in March 2005, only an estimated 5.1% of people in need of ART were accessing ART [5]. The equivalent rate amongst children was 3.1%, suggesting that children may face even bigger barriers to ART access than adults (*ibid*.). Despite this proportion has now increased, literature and key informant interviews suggest that barriers still exist and are related in part to ART sites not reaching their full capacity to provide treatment for children, the need for longer and more complex consultation procedures and related opportunity costs, transport costs and stigma. Strategies to enhance children's ART access became an area of critical advocacy in Malawi as part of the global WHO/UNICEF children and AIDS campaign [6,7].

Understanding ways to promote children's adherence to ART is also vital. In adults, 95% adherence is necessary to reduce the development of drug resistance and for drugs to work effectively in prolonging life [8]. Likewise, non-adherence is a predictor of rapid disease progression [9]. Studies, which examine ART adherence among children are largely from developed world settings, utilize a variety of measurements and associate adherence with immunological and virological markers [10-13]. Studies from low and middle-income countries were recently reviewed and the lack of cultural adaptation of tools to measure adherence as well as the lack of formative qualitative research has been identified as a gap [14].

Measuring adherence to ART is fraught with difficulties and there is no gold standard. Direct measurement approaches identified in the literature include observation [15], and assessment of drug levels [16,17]. Indirect measurement approaches include pill counts [18-20], patient reported intake [12,21], physician's assessment [13], number of prescriptions filled [10] and electronic monitoring, using for example the Medication Event Monitoring System (MEMS), whereby the opening of bottles of drugs is electronically recorded [13,22]. Measuring adherence is further complicated amongst children because some of these methods are not appropriate, for example the pill count is complicated by different formulations (the use of syrups) and varying dosage dependent on weight and body surface [23]. There are also additional challenges in resource-poor contexts and it has been argued that in Africa adherence will not be as good as in settings, which can afford resource-intensive programmes [24]. However, evidence is increasing that adherence of children in low and middle income countries might be equal if not better than in high income countries, where most studies have shown adherence rates of less than 75% [14]. For example, good adherence using a simple tool like measuring pills returned to the clinic was correlating with viral load suppression in Cape Town, South Africa [25]. In another study with good clinical and immunological outcomes, adherence was measured by caregiver's and child's self report [26]. However, further research on adherence in African countries is urgently needed [27] and results might be different in rural settings [28]. The contribution of factors like disclosure status of the child is still controversial [29,30].

This article merges findings from two complementary studies (one quantitative, one qualitative) conducted at the Lighthouse in urban Lilongwe, Malawi, with the aim to contribute to the debate on developing strategies to support ART adherence amongst children in resource-poor contexts. The objectives are (1) to describe the characteristics and outcomes of children on ART (*quantitative*) (2) to describe children's adherence according to scheduled appointments and caregivers' report of ART intake (*quantitative*) (3) to explore caregivers' perceptions of barriers and enabling and motivating factors to adhering to ART for children (*qualitative*).

## Methods

### Study setting

The Lighthouse Clinic is located in urban Lilongwe, Malawi, at the campus of the central hospital. Lighthouse, a charitable trust since 2001, functions as a part of the public health sector and provides care for people affected by and infected with HIV/AIDS. Services include HIV testing and counselling, home based care and clinical care, including – since July 2002- ART. Public health services in Malawi are offered free at the point of delivery, and ART has been provided free since June 2004. It is important to note that the studies took place when patients had to pay for treatment and a month's supply of paediatric formulation (depending on weight band and drugs used) ranged from USD14 to 43.

Lighthouse has standardized procedures for visits, including initial visits, follow-up visits and initiation of ART for children. For each child initiating ART, a master-card with name, age, ID number and scheduled visit dates is prepared and kept in a separate folder and reinforces information recorded in a child's health passport or treatment card. Before starting ART, the caregiver and the child (if the diagnosis was disclosed) attend a one-hour mandatory education session.

### Description of weight-band based ART regimens used during the study period

The initial regimen used for children at Lighthouse was AZT, 3TC and NVP (see additional file 1: ART- regimen a). Several formulations were used, at different prices: generic AZT syrup, AZT 100 mg capsules, generic 3TC syrup, generic 3TC 150 mg tablets, generic NVP syrup and 200 mg NVP tablets. A limited number of 3TC 150 mg tablets and AZT 100 mg capsules were free of charge during the study period through donations. According to patients' body weight and body surface tablets had to be split into halves and commercial pill cutters were supplied. The first edition of the national ART guidelines [31] recommended d4T, 3TC and NVP for both adults and children as first line ART regimen. From January 2004 onwards, new children started on a combination of generic dual combination tablets of d4T/3TC and single generic NVP tablets (see additional file 2: ART- regimen b). When they reached 25 kg body weight they started or switched to adult fixed dose combination (FDC) of d4T 30 mg/3TC/NVP (Triomune 30) 1 tablet twice daily. By July 2004, only 3 of 29 children were still on their initial regimen of AZT/3TC and NVP.

### Quantitative methods

All children less than 13 years of age who started ART at the Lighthouse between 1^st ^September 2002 and 31^st ^January 2004 were enrolled in the study and observed until 31^st ^July 2004. The basic characteristics of children on ART and outcome measures were assessed through analysis of the master card at baseline and at the end of the study period. After a child's initial visit to the clinic, a main caregiver was identified and an appointment for the weekly paediatric clinic was given. Eligibility for ART treatment was defined immunologically and clinically using WHO stages I to III according to WHO guidelines [32]. In addition, caregivers were informed about the cost of the treatment and children started only if caregivers confirmed that they could afford ART. However, no detailed assessment of caregivers' ability to afford ART was done. Prior to ART initiation the caregiver received an information leaflet. After initiation of ART children were given appointments after 2 weeks and thereafter visited the clinic at monthly intervals. During each ART visit caregivers were asked to report missed ART doses within the last 3 days prior to the visit according to the format below:

Is your child on long-term treatment (like ARV, CPT, IPT, TB tx)? Y/N, if yes, specify ____________________

How many doses did he/she miss yesterday? _______

The day before that? __________

The day before that? __________

When was the last time your child missed a dose of medication? _________

What keeps him/her from taking the medication? (If your child is on ARV, the question above relates to ARVs. Otherwise indicate to which other long-term treatment it relates)

Appointments were monitored from the child's first visit at the Lighthouse. Once on ART, each caregiver's report of their children's ART intake was recorded for at least 6 months. All quantitative measures were entered into an MS Excel spreadsheet and regularly updated.

### Qualitative methods

To complement the quantitative data, a qualitative study was conducted in June and July 2004, when free ART and an increased uptake of ART was imminent. Two complementary qualitative methods were used: focus group discussions (FGD) and critical incident narrative (CIN), a form of in-depth interview, which trace a particular illness-experience through time (in this case the pathway to initiation of ART and experiences since then). During 4 FGDs, each with 6 to 8 female caregivers, barriers for children to adhere to ART were explored. In 5 CINs, experiences of different female and male caregivers of HIV-infected children on ART, and of children who were lost to follow-up or interrupted treatment, were examined.

Qualitative data collected from the FGDs and CINs were transcribed verbatim. Following the framework approach to qualitative analysis [33] the FGD and CIN transcripts were analysed with codes to identify key themes emerging and comparing and contrasting the views and experiences of different respondents.

### Ethics and quality assurance

The Malawian National Health Sciences Research Committee approved both studies. In addition, the quantitative study was approved by the London School of Hygiene and Tropical Medicine ethics committee, UK, and the qualitative study by the Liverpool School of Tropical Medicine ethics committee, UK. Quality was assured in the quantitative study through regular audit of the patient's file and comparison against entries in the excel spreadsheet, and in the qualitative study through the skills of a senior social scientist with experience of conducting qualitative research on HIV in Malawi (IM).

## Results

### Quantitative results I: Characteristics and outcomes of children on ART

Table 1 shows the baseline characteristics of the 47 children as they started ART. Their median time on ART was 33 weeks (range 2–91).

**Table 1:** Baseline characteristics of children included in the quantitative study

Total number of patients		47
Male		24

Female		23

Age at start of ART (months)	Median (range)	98 (8–151)

WHO stage	II	22

	III	25

Immunological stage	I	1

	II	8

	III	36

	Not classified	2

The cumulative outcomes as of 31^st ^July 2004 were as follows: 36 children were alive and on ART, 5 were lost to follow-up (not seen in the clinic for 3 months after picking up their last one-month supply of ART), 3 children died and 3 children discontinued ART. Of those who discontinued, one child started d4T/3TC and NVP and developed abdominal distension with hepatomegaly and ascites after 8 weeks on ART. Lab investigations were not available through the central hospital. The presumptive diagnosis of NVP- associated hepatitis was made and ART had to be permanently stopped since no alternative triple combination was available. The two other children had started on AZT, 3TC and NVP but stopped because caregivers could not afford to continue ART after 12 and 4 months, respectively. One child continued to be in follow-up and re-started when ART became free, but outside the study period.

### Quantitative results II: Strategies to assess adherence

#### Scheduled appointments

In total, 451 appointments for the 47 children before and on ART were recorded. Due to incomplete records (no date of visit entered in the visit form, no appointment given), 401 were available for analysis (Table 2).

**Table 2:** Adherence with scheduled appointments (including visits while not on ART)

Total no. of visits with appointments given (%)	401 (100)
As scheduled	206 (51)

Before appointment	58 (15)

Within 1 week after appointment	63 (16)

More than 1 week after appointment	74 (18)

More than 80% of all visits (327/401) were either as scheduled, before the agreed day or within a week (the paediatric clinic is once a week). Fifteen out of the 47 children never came more than one week late, 9 came more than a week late once and 12 came more than a week late twice. Thirty-one out of 47 patients made visits before their agreed appointment, and the most common reasons for coming earlier were acute illnesses, anticipated side effects, and forgetting the appointment date.

#### Self report

Once on ART, caregivers were asked at each visit whether doses had been missed in the previous 3 days. In total, 441 ART-visits were recorded. Complete information was available for 363 visits. Thirty-four out of 47 children never missed a single ART dose within the last 3 days according to the caregivers' report. Visits of these children account for 73% of all visits available for analysis. Caregivers of 13 children, accounting for the remaining 27% of all visits, reported missing doses once or twice during 17 visits. The following reasons for missed doses were given during these 17 visits: no drugs at home for different reasons-5 (no stock in pharmacy- 1, no money to buy- 2, no specific reason given- 2), mother went to work early – 2, travelling-2, child was hospitalised and semiconscious due to an road traffic accident- 1, child was vomiting- 1, had visitors- 1, no reason given- 5. Seven out of these 13 patients with missing doses did not visit the clinic at the agreed date the day they reported the missing and 6 visited on time. These 7 patients made 9 visits with reported missed doses: 6 visits were within a week after the appointment, 2 visits more than a week after the appointment, and 1 visit was before the appointment.

Thirty-two patients visited the clinic at least once within a week after the actual appointment date. Out of those, 27 patients had delayed visits while on ART. Twenty-one reported not having missed any ART dose within the 3 days prior to their (delayed) visit, 4 patients reported during at least one delayed visit to have missed a dose, and for delayed visits of 2 patients on ART there was no adherence information available. Therefore, not more than 6 out of 27 patients with delayed ART visits up to one week reported to have missed doses during their delayed visits. Twenty-two out of 36 children alive and on ART by the end of July 2004, were fully adherent according to caregivers' reports throughout the study period. They never reported to have missed any dose over the past three days and they never mentioned, that they had missed a dose in response to the question "When was the last time your child missed a dose of medication?"

### Qualitative results: caregivers' understanding of ART and perceived barriers and enablers for adherence

#### 'Assisting soldiers in the body': Understanding and administering ART

In general caregivers showed a good understanding of how ART works. Many respondents referred to ART as 'bringing back immunity', 'adding to immunity' or 'assisting soldiers in the body who fight with illnesses'. Most caregivers understood that ART had to be taken on a regular basis for life, for example:

*'It is through the child's life because you want the immunity of the child to be at a good level so that the virus should not multiply itself a lot and if he stopped taking them the virus might get used to the medicine and start to multiply again' *(CIN, female caregiver)

However, one female caregiver understood that children stop taking treatment when they reach 15 years of age. Caregivers were able to explain that dosage was dependent on the child's age and some female caregivers also made the association with weight. Most respondents reported that it was the mother or female caregiver who gave the drugs to the child and also accompanied the child to the Lighthouse. One caregiver of a child who had defaulted from treatment reported that the child took the drugs on his own as he was staying in the village with his grandmother. Most caregivers reported that they did not face major challenges in children taking drugs. This was mainly attributed to the fact that the child had been sick for a long time and was used to taking drugs. However, one woman explained that a child she was caring for would keep the drugs in his mouth for a long time and spit them out later. All respondents mentioned that children suffered from side effects of ART. Common conditions attributed to the ART and classified by the caregivers as side effects were: rash, stomach pains, leg swelling and pain, diarrhoea, coughing, vomiting, and yellow eyes.

#### 'He is able to play': Enablers for adhering to ART

Positive visual and physical changes emerged as the key-motivating factor in supporting children to adhere to ART.

*'I feel that there has been a great change because at the time he was starting, he had low weight and his stomach was big but after he started he changed and he was not very sick. Now he has changed, after the medicine he is able to play' *(CIN, female caregiver to a child on ART for 12 months)

It was also generally felt that ART reduced the time that caregivers had been spending with a child admitted to hospital, as well as the time lost from economic activities as explained in the quotes below.

*'She is not getting as sick as before and since she started treatment she has not been admitted to hospital' *(CIN, female caregiver to a child on ART for 13 months)

*'The child's frequent illness usually affected us, as our assistance is required and if we do not have money, we have to find some and we also have to bring the child to the hospital which means we cannot go to work and therefore also affects our home' *(CIN, male caregiver to a child starting ART)

Love and commitment to the child also emerged as key themes and were important in supporting the child to adhere to ART. Female caregivers in both the FGD and CINs highlighted the importance of good planning in supporting adherence, such as giving the drugs before a mother goes to work, and ensuring that the child does not run out of drugs. Women also reported that the children themselves sometimes reminded them if they forgot to give them the drugs.

#### Multiple challenges: Barriers to adhering to ART

Cost stood out as the key barrier and caused children to attend late to appointments and to miss doses. The caregivers of children from orphanages feared that the children would run into problems if at any time sponsors would stop giving money for ART. Some respondents also explained that caregivers considered deliberately reducing the doses to make the drugs last longer. Some participants were living outside of Lilongwe and had to travel long distances to come to the Lighthouse. As a result, cost of transport and time spent for travelling increased. One respondent, whose child had been lost to follow-up and was traced during the study, explained that the child had constantly missed drugs because he was living with his grandparents. They had not been supervising him in taking treatment and had left the child with the responsibility of taking his drugs as required. The respondent also reported that the child stayed with the grandparents in Rumphi (400 kilometres from Lilongwe) and at the time the child needed to go to the Lighthouse, they did not have money for transport.

*'When I went to attend the funeral I asked his granny because I saw that the drugs were finishing to help me with money [so she could pick the child to get his drugs] but she refused saying that she did not have money. The problem for me is that my husband died and I stay by myself and it is difficult to find money.' *(Female caregiver, child lost to follow-up)

Multiple responsibilities of caregivers were identified as another barrier. Children of caregivers who worked or performed other activities outside their homes, or who delegated the responsibility of administration of drugs to housemaids or other household members, were at higher risk to miss doses.

*'When I had to leave early for work I could leave his father to give him the drugs but his father could forget to do so' *(CIN, female caregiver to a child on ART for 12 months)

Multiple responsibilities were a particular constraint for caregivers at orphanages as illustrated by the following quote:

*'What sometimes happens is that I look after many children and sometime I get very busy as I am looking after 11/12 children, so maybe sometime something sudden can happen. May be one of the other children is ill or has been injured and you can get distracted by these other things but still if it is evening and you forgot you have to wake the child up and give them the drugs' *(CIN, female caregiver to a child on ART for 11 months)

Other barriers that emerged were changes of the routine, for example giving responsibility to someone else to give the drugs if the main caregiver is away, for example attending a funeral. This was further complicated by stigma and fear of disclosure and some participants faced difficult decisions about whom to ask to support drug adherence amongst children in their absence. Death of caregiver was highlighted as a particular challenge to adherence. A male caregiver reported that his child was missing cotrimoxazole preventive therapy (CPT) because he had left his sister to give the drugs to the child. Travelling and visiting relatives could also cause problems in adherence to drugs. In some cases, the responsibility for drug taking was left largely to the child:

*'The problem could be that he looks after himself but he is still a child and of course sometimes his granny reminds him but you know sometimes they can go to attend a funeral and the child ends up being alone at home' *(CIN, female caregiver to a child lost to follow-up)

One respondent stated that once a child started to feel better, the caregiver might feel less inclined to insist that the child takes ART on a regular basis. System-related barriers were also cited, such as the Lighthouse running out of drugs and long waiting times.

## Discussion

According to the caregivers' reports 72% (34/47) of children never missed a single ART dose, suggesting that 34 children had complete (100%) adherence over the full study period for the 3 days prior to each visit. More than 80% of all children's visits occurred within a week of the agreed day. It appears that relatively high levels of adherence confirmed by these two different quantitative findings are further corroborated by favourable major clinical outcomes in this group of children with advanced HIV disease (77% alive and responding to treatment after a median treatment duration of 33 weeks). However, interpretation of these findings needs to be treated with caution. First, caregivers' report has a low sensitivity to detect poor adherence [25]. We did not have the means to perform more objective measurements, like drug plasma levels, or viral load tests because of cost. We could not use pill counts as a measure because we used pills, capsules and syrups concurrently. Monitoring pharmacy refills was difficult because caregivers bought ART for their children at different pharmacies at the central hospital or in private pharmacies in town. Second, our sample size is small making interpretation of clinical outcomes challenging. The sample size reflects the reality of the limited number of children who were on ART at the time of study: the 47 children represent the complete cohort of children who started ART at Lighthouse within this period. However, outcomes in other studies from Malawi are similar [34,35]. Third, a comparison with adherence reported by others is difficult; tools used to measure adherence are heterogeneous and poorly validated [14]. Exact provision of a one-month supply of anti-retrovirals (ARVs) is difficult if different formulations (split tablets and syrups) are used. ARVs remaining from previous supplies or missed doses during time periods prior to the 3-day recall can allow patients to report complete adherence at a delayed visit or can result in over-reporting of adherence with ARVs. The importance of regularly asking questions about missed doses might lie more in reminding and motivating caregivers to support adherence than actually measuring adherence. Providers should emphasize on taking full doses and not pill sharing (as mentioned in an FGD) in ongoing patient communication.

The complementarity of multi-method research is well recognised in the literature, with findings from qualitative research helping to interpret and contextualise quantitative results [36-38]. In our research, the qualitative study clearly highlights that caregivers are well motivated to support children in adhering to ART, despite the multiple challenges this presents. Seeing a child become much healthier after taking ART is a strong adherence motivator, despite the perceived side effects. A positive treatment response strongly reinforces adherence [39]. If ART is started too early it is possible that negative side effects will outweigh the perceived positive effects of ART with implications for adherence.

The studies took place within a clinic with well-trained and well-motivated staff at the time when patients had to pay for ART [40]. The ART regimens used were complex depending on the particular situation of the patient and drug availability. The situation at the Lighthouse is now remarkably different and should arguably further reinforce adherence. The regimen currently used, in line with the national ART scale-up policy, is simpler, easier to procure and always available. With funding from the Global Fund, ART is now provided free, meaning that patients no longer face the key adherence barrier of cost of drugs. This is an extremely positive step, which has the potential to enhance access to ART for poor adults and children alike [5]. However, free drugs had meant increased demand for ART and waiting times were prone to being much longer than they used to be. Anecdotal evidence highlights that this had led to patient dissatisfaction and possibly defaulting from care [41].

By March 2005, 34 public health facilities provided ART; 886 children in 13 of these sites (<13 years) had registered for ART, representing 5% of the total cohort [42]. ART is now available in 170 public sites throughout Malawi and 16,600 children (<15 years) have started ART in the public sector, representing 8% of the total cohort [4]. However, many sites face severe human resource shortages [43] resulting in long waiting times, also confidence and expertise in paediatric HIV care is still limited. Results of this study have fuelled the discussion about user fees for ART in Malawi [44] and contributed to debates on how to address the urgent need to support complementary, user-friendly approaches to supporting adherence in children, who have potentially a whole lifetime of drug-taking ahead of them. A paediatric ART flip chart was developed through a similar process as the adult ART flip chart and rolled out in the country [45]. We hope this tool will contribute to sustaining the paediatric 1^st ^line regimen longer since second line drugs are only available in few sites, can be more complex, more difficult to adhere to and more expensive.

International guidelines still rely largely on western concepts with little contribution of qualitative research to address cultural context and overburdened health facilities to support children's adherence to ART in resource-poor contexts [14,46]. This narrow focus is not appropriate. The findings from the 2 studies presented here clearly show the need for contextual understandings and holistic responses that are grounded in health workers', caregivers' and children's experiences, and the challenges and priorities in resource-poor contexts. Malawi is now addressing adherence with paediatric ART [47,48].

The need for a holistic approach is supported by research on adherence amongst children and adolescents in resource-rich countries which highlights the importance of focusing on patient characteristics and the 'triadic nature of the doctor-caregiver-child relationship' [49]. Treatment outcomes are dependent on adherence, which in turn is dependent on a complex array of systems, individual and socio-cultural related barriers. In resource-poor contexts, system-related factors appear to be strong determinants of adherence. It is imperative for children to be able to stay as long as possible on first line ART in resource-poor contexts, such as Malawi. Hence these multiple factors need to be addressed in a holistic approach in order to maximise chances of supporting children's adherence to ART in resource-poor countries.

## Conclusion

It is possible for children in resource-poor contexts to access and adhere to ART. The launch of the WHO/UNICEF global initiative on children and AIDS has pushed this agenda forward [6]. Quantitative and qualitative insights from the Lighthouse in Lilongwe have shown that caregivers and children are well motivated to adhere despite the presence of both individual, familial and health-system related barriers. User-friendly pragmatic structures can be put in place (such as caregivers' report, appointment monitoring and pill count measurements) to monitor, assess and support adherence in challenging resource-poor contexts. In supporting adherence there is a need for a holistic and contextually bound approach that encompasses the priorities of health providers, caregivers and children.

## Competing interests

The authors declare that they have no competing interests.

## Authors' contributions

RW and IM managed the study, prepared the manuscript and participated in data collection and analysis. RW, IM and ST designed the study. ST contributed to data analysis and reviewed the manuscript. JN, SP and DC contributed to data collection and reviewed the manuscript. All authors read and approved the final manuscript.

## Pre-publication history

The pre-publication history for this paper can be accessed here:



## Supplementary Material

Additional file 1 **Weight-band based dosage table for AZT, 3TC and NVP**. 100 Malawian Kwacha (MK) = 1 USD. Click here for file

Additional file 2 **Weight-band based dosage table for adult dual fixed dose combination (FDC) of d4T/3TC and NVP**. 100 Malawian Kwacha (MK) = 1 USD; LamS30 or LamS40 = dual FDC of either d4T 30 mg or d4T 40 mg and 3TC 150 mg; Triomune 30 = FDC of d4T 30 mg/3TC 150 mg/NVP 200 mg. Click here for file
