# Supplementary material for: Supporting children to adhere to anti-retroviral therapy in urban Malawi: multi method insights
Source: BMC Pediatr. 2009 Jul 14;9:45. doi: 10.1186/1471-2431-9-45 (PMC2717958; doi:10.1186/1471-2431-9-45)
Supplement: Additional file 1 — Weight-band based dosage table for AZT, 3TC and NVP. 100 Malawian Kwacha (MK) = 1 USD. [file 1471-2431-9-45-S1.pdf]

body weight	AZT BD		3TC BD		NVP OD (first two 2 weeks only)		NVP BD		Price/mth.
(kg)	300mg tabl	syrup 10mg/ml	150mg tabl	syrup10mg /ml	200mg tabl	syrup 10mg/ml	200mg tabl	10mg/ml	(MK)
3- <4		4		1,5		3		5	**1385**
4- <6		6		2,5		4		6,5	**1971**
6- <8	1/4			3		5		8	**1938**
8- <10	1/4			4		6		9	**2220**
10- <12	1/4	2		5		7	1/2		**2226**
12- <14	1/4	4		6		8	1/2	2	**2883**
14- <16	1/4	5		7		9	1/2	3	**3282**
16- <20	1/2		1/2		1/2		1/2	5	**3005**
20- <24	1/2	2	1/2	2	1/2		1/2	7	**3803**
24- <28	1/2	3	1/2	4	1/2		1		**4115**
28- <30	1/2	4	1/2	5	1/2		1		**4373**
