# Supplementary material for: Supporting children to adhere to anti-retroviral therapy in urban Malawi: multi method insights
Source: BMC Pediatr. 2009 Jul 14;9:45. doi: 10.1186/1471-2431-9-45 (PMC2717958; doi:10.1186/1471-2431-9-45)
Supplement: Additional file 2 — Weight-band based dosage table for adult dual fixed dose combination (FDC) of d4T/3TC and NVP. 100 Malawian Kwacha (MK) = 1 USD; LamS30 or LamS40 = dual FDC of either d4T 30 mg or d4T 40 mg and 3TC 150 mg; Triomune 30 = FDC of d4T 30 mg/3TC 150 mg/NVP 200 mg. [file 1471-2431-9-45-S2.pdf]

| bwt (kg) | Dose | | cost/month (MK) |
|---|---|---|---|
| | AM | PM | |
| 8-<10 | 1/4 LamS 40 + 1/2 NVP 200 | 1/4 LamS 40 + 1/4 NVP 200 | 1076 |
| 10-<15 | 1/2 LamS 30 + 1/2 NVP 200 | 1/2 LamS 30 + 1/2 NVP 200 | 1701 |
| 15-<20 | 1/2 LamS 40 + 1 NVP 200 | 1/2 LamS 40 + 1/4 NVP 200 | 1926 |
| 20-<25 | 1 LamS 30 + 1 NVP 200 | 1/2 LamS 30 + 1/2 NVP 200 | 2552 |
| ≥25 | 1 Triomune30 | 1 Triomune30 | 2500 |
